# Engineering Au Nanoclusters for Relay Luminescence Enhancement with Aggregation-Induced Emission

**DOI:** 10.3390/nano12050777

**Published:** 2022-02-25

**Authors:** Pei Zhou, Nirmal Goswami, Tiankai Chen, Xiaoman Liu, Xin Huang

**Affiliations:** 1 School of Environmental and Municipal Engineering, North China University of Water Resources and Electric Power, Collaborative Innovation Center for Efficient Utilization of Water Resources, Henan Key Laboratory of Water Environment Simulation and Treatment, Zhengzhou 450046, China; 2 MIIT Key Laboratory of Critical Materials Technology for New Energy Conversion and Storage, School of Chemistry and Chemical Engineering, Harbin Institute of Technology, Harbin 150001, China; liuxiaoman@hit.edu.cn; 3 Materials Chemistry Department, CSIR-Institute of Minerals and Materials Technology, Acharya Vihar, Bhubaneswar 751013, India; ngoswami@immt.res.in; 4 Department of Chemical and Biomolecular Engineering, National University of Singapore, 10 Kent Ridge Crescent, Singapore 119260, Singapore; chen.tiankai@u.nus.edu

**Keywords:** luminescent gold nanoclusters, aggregation-induced emission, relay luminescence enhancement, thermosensitive polymer, self-assembly

## Abstract

The research of aggregation-induced emission (AIE) has been growing rapidly for the design of highly luminescent materials, as exemplified by the library of AIE-active materials (or AIEgens) fabricated and explored for diverse applications in different fields. Herein, we reported a relay luminescence enhancement of luminescent Au nanoclusters (Au NCs) through AIE. In addition, we demonstrated the emergence of reduced aggregation-caused luminescence by adjusting the temperature of the Au NC solution. The key to induce this effect is to attach a thermosensitive polymer poly(N-isopropylacrylamide) (PNIPAAm) on the surface of Au NCs, which will shrink at high temperature. More interestingly, the as-synthesized Au NCs-PNIPAAm can self-assemble into vesicles, resulting in an obvious decrease in the luminescence intensity in aqueous solution. The combination of relay luminescence enhancement (by AIE) and luminescence decrease (induced by thermosensitive polymers) will be beneficial to the understanding and manipulation of the optical properties of Au NCs, paving the way for their practical applications.

## 1. Introduction

Thiolate-protected gold nanoclusters (Au NCs, <2 nm) have witnessed dramatic growth in both basic research and practical applications [1,2,3,4,5], mainly due to their unique physical and chemical properties, such as HOMO-LUMO transitions, quantized charging, molecular magnetism, molecular chirality, and photoluminescence [6,7,8,9]. In particular, the fabrication of luminescent Au NCs has become an interest of research in the NC community as Au NCs not only show strong luminescence but also feature low toxicity and good biocompatibility [10,11,12,13,14,15]. Due to the intriguing physicochemical properties, luminescent Au NCs find increasing acceptance in various applications in sensing (as luminescent probes for chemical sensors and biosensors), bioimaging, and light-emitting diodes (LEDs) [16,17,18,19,20,21,22]. Luminescent Au NCs are usually synthesized by reducing Au(I)-thiolate complexes with strong reducing agents, such as sodium borohydride, NaBH_4_. However, these Au NCs often exhibit weak luminescence and their quantum yields (QYs) rarely exceed 0.1% [23]. A technical challenge in this field is therefore to synthesize Au NCs with strong luminescence, which has been partially addressed by some recent studies in developing efficient synthesis methods for luminescent Au NCs [12,24,25,26,27,28,29,30,31,32,33,34].

For example, our group developed a thermal reduction method to produce thiolate-protected Au NCs with a QY of ~15% [35]. The strong luminescence of the thiolate-protected Au NCs is attributed to the aggregation of Au(I)-thiolate complexes onto the Au(0) core. This aggregation-induced emission (AIE) phenomenon is constructive, because when aggregated in solution, non-luminescent molecules can generate strong luminescence, and the degree of aggregation can be used to control the intensity of luminescence [36,37]. Importantly, the implementation of AIE effect on cluster luminescence is rather a recent entry in the NC field. In addition, recently our group and several other groups identified some AIE-type metal NCs, most of which show a higher QY than the conventional thiolate-protected Au NCs [38,39,40,41]. In fact, the two key factors leading to the AIE effect are restriction of intramolecular rotation (RIR) and intramolecular vibration (RIV), which are well-demonstrated in organic AIEgens [42,43,44], and they are also applicable to thiolate-protected Au NCs. Although recent progress has been made in the synthesis of AIE-type Au NCs, the mechanism of the AIE effect on NC luminescence is not yet fully understood. In addition, it is still challenging to finely control the luminescence properties of AIE-type metal NCs by modifying their surface structure.

Generally, AIE-active molecules consist of a number of free rotors and vibrators, such as phenyl in a typical propeller-like non-planar molecule, hexaphenylsilole (HPS) [28,29]. Once the rotation and vibration of these rotors and vibrators are greatly restricted, that is, they are unable to rotate or vibrate freely in a dilute solution, a strong AIE effect will occur. The aggregation behavior can be induced by several physical, chemical, and engineering strategies. For example, temperature or pressure can be used to construct the aggregated materials through physical strategies [45,46,47]. In a typical chemical strategy, weakly polar solvents can be used to adjust the viscosity and polarity of the solution. By applying these strategies, various AIE materials have been successfully designed for applications in bioimaging [48,49,50], biomedicine [51], and sensors [52]. In another type of luminescent material, the luminescence intensity can also be reduced at high concentrations (forming aggregates), which is attributed to a distinctly different mechanism called aggregation-caused quenching (ACQ) [44]. Many conventional luminescent molecules and luminophores, such as planar luminophoric molecules and quantum dots [53,54], inherit this ACQ effect [55]. In view of the expected AIE and undesirable ACQ effects, we hypothesized that luminescent Au NCs instead of conventional non-luminescent molecules can contribute relay luminescence enhancement toward the AIE effect, which may be of interest to the undiscovered optical properties of metal NCs. Researchers from various fields are motivated to further decipher the AIE mechanisms [56,57]. More interestingly, by attaching polymer poly(N-isopropylacrylamide (PNIPAAm) as an additional ligand to the Au NC surface, the as-grafted thermosensitive polymers can be designed to shrink at high temperature, which may affect the luminescence of the AIE-type Au NCs. Another interesting question is whether the as-generated Au NCs-PNIPAAm can also show relay intensity decrease after the relay luminescence enhancement (due to the AIE effect). Exposing this multifunctional Au NC derivative in a programmed relayed mode will further encourage us to understand the undiscovered optical properties of Au NCs.

**Scheme 1:**
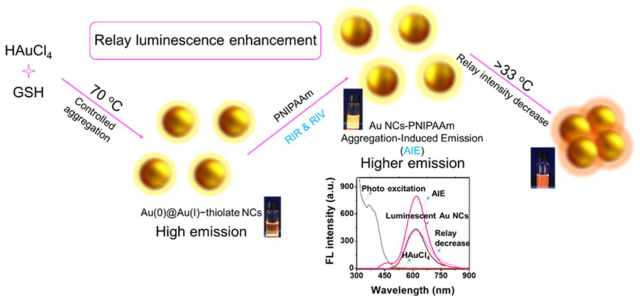
Schematic illustration of the synthesis of luminescent Au NCs and Au NCs-PNIPAAm conjugates, showing the relay luminescence enhancement with AIE characteristics and relay intensity decrease with thermosensitive properties.

Under the guidance of the design strategy of relay enhancement with AIE characteristics and decrease of luminescence intensity with temperature, we can now control the aggregation of Au(I)-SG (SG denotes glutathione) complexes and PNIPAAm to generate relay luminescence enhancement. We first prepared a luminescent Au NC that exhibits strong luminescence at 610 nm (λ_ex_ = 365 nm) with a QY of ~15%. Subsequently, PNIPAAm was used to construct Au NCs-PNIPAAm conjugates, where an end-capped mercaptothiazoline-activated PNIPAAm reacted with the primary amine group of GSH on the surface of Au NCs. The resulting conjugates showed a strong relay luminescence enhancement, which is much higher than the original luminescent Au NCs without aggregation at room temperature. However, above the lower critical solution temperature (LCST) of the thermosensitive polymer (e.g., 32 °C), the solution showed weak luminescence, which was induced by the aggregation of the Au NCs-PNIPAAm conjugates at a high temperature. Therefore, we attribute the strong relay enhancement in luminescence of the NC conjugates in water to the restriction of RIR and RIV associated with the extended PNIPAAm chains in the non-aggregated state. In addition, the well-defined Au NCs-PNIPAAm conjugates can self-assemble into vesicles in aqueous solution, which can guide the transition from AIE to relay luminescence intensity decrease by adjusting the solution temperature (Scheme 1). These two aspects related to the luminescence of Au NCs provide a good platform to design high-quality luminescent metal NCs.

## 2. Experimental Materials and Methods

### 2.1. Materials

Hydrogen tetrachloroaurate trihydrate (HAuCl_4_·3H_2_O) and L-glutathione in the reduced form (GSH) were obtained from Sigma-Aldrich. N-isopropylacrylamide (NIPAAm, Aldrich, St. Louis, MO, USA, 98%) was recrystallized twice in hexane and toluene prior to use. 2,2′-Azobis-(isobutyronitrile) (AIBN, Sigma, St. Louis, MO, USA, 98%) was recrystallized from methanol. 2-mercaptothiazoline (Sigma, 98%), acetonitrile (CH_3_CN, 99.5%, Aladdin Industrial Corporation, Shangai, China), and glutaraldehyde (GA, Aladdin, AR, Shangai, China) were used as received. Ultrapure water (Milli-Q, Millipore, Burlington, MA, USA) with a resistivity of 18.2 MΩ was used as the solvent throughout the study. All other reagents and solvents are of analytical grade and can be used directly without further purification.

### 2.2. Characterization Methods

UV-visible absorption spectra were recorded using a Shimadzu UV-1800 photospectrometer (Shimadzu, Kyoto, Japan). Experiments were carried out in a 1.5 mL semi-micro quartz cuvette with a 1 cm optical path. Fluorescence spectra were recorded on a PerkinElmer LS-55 fluorescence spectrometer (PerkinElmer, Waltham, MA, USA). Surface potentials of Au NCs and Au NCs-PNIPAAm were measured in aqueous solutions on a Malvern Zetasizer (Zeta) Nano-ZS equipment (Malvern Instruments, Malvern, UK), and *ζ*-potential was obtained using the Smoluchowski relation. The average particle size and size distribution of the NCs (0.2 mg mL^−1^, pH 6.8) were characterized by dynamic light scattering (DLS) on a Malvern Zetasizer Nano-ZS at a fixed scattering angle of 90°, after being filtered by 0.45 μm Millipore filters. ^1^H NMR spectra were recorded on Bruker Advance-400 MHz spectrometer (Bruker Corporation, Ettlingen, Germany), with CDCl_3_ as solvent at room temperature. Chemical shifts (δ) were expressed in ppm. X-ray photoelectron spectroscopy (XPS) measurements were performed on a VG Escalab MKII spectrometer (VG Scientific, Waltham, MA, USA). Thermogravimetric analysis (TGA) was conducted on a Shimadzu TGA-60 analyzer (Shimadzu, Japan) under N_2_ atmosphere (flow rate of 20 mL min^−1^). Photoluminescence lifetimes were measured by time-correlated single-photon counting (TCSPC) on a Horiba Jobin Yvon Fluorolog-3 spectrofluorometer (Horiba Scientific, Kyoto, Japan) with a pulsed light-emitting diode (LED) (344 nm, pulse duration <1 ns) as the excitation source. Optical and fluorescence microscopy was performed on a Nikon TS100 manual inverted fluorescence microscope at 100× magnification (Nikon, Tokyo, Japan). Confocal laser scanning microscope (CLSM) images were obtained with a Nikon A1 laser scanning microscope equipped with 100× oil immersion objective. A drop of hollow vesicles suspension was added to Lab-Tek chamber (Electron Microscopy Sciences, Hatfield, PA, USA) (Nikon, Japan), which was then filled with ultrapure water, and the vesicles were allowed to settle down. Transmission electron microscopy (TEM) images were taken on a JEOL JEM 2010 microscope operating at 200 kV. Atomic force microscopy (AFM) images were obtained using a Bruker Dimension ICON (JEOL, Tokyo, Japan). Silicon wafers were cleaned with DI water and dried with a stream of nitrogen. Thickness and roughness of the vesicles were determined as half of the height of the collapsed flat regions of dried vesicles from the height histograms using NanoScope analysis software (Bruker Corporation, Germany). The pH value was measured using METTLER TOLEDO Seven Compact meter (Mettler-Toledo, Greifensee, Switzerland).

### 2.3. Synthesis of Luminescent Au NCs

Freshly prepared aqueous solutions of HAuCl_4_ (20 mM, 1 mL) and GSH (100 mM, 0.3 mL) were mixed with 8.7 mL of ultrapure water at 25 °C. The reaction mixture was heated to 70 °C under gentle stirring (500 rpm) for 24 h. An aqueous solution of strongly orange-emitting Au NCs was obtained.

### 2.4. Synthesis of End-Capped Mercaptothiazoline-Activated PNIPAAm by RAFT Polymerization

The modification of mercaptothiazoline-activated activated PNIPAAm was according to the reported method. For the mercaptothiazoline activation, briefly, mercaptothiazoline-activated trithiol-RAFT agent (18.9 mg, 50 μmol), AIBN (1.7 mg, 10 μmol), NIPAAm (847 mg, 7.5 mmol), and acetonitrile (8 mL) were added to a 25 mL of round-bottom flask. The flask was then sealed and the solution was degassed via four freeze pump-thaw cycles. The polymerization was carried out at 60 °C for 8 h (conversion 45%), and the resulting product was purified by three times precipitation in diethyl ether/hexane (2:1 volume ratio). The as-obtained polymer was characterized by ^1^H NMR spectroscopy in CDCl_3_. The proton signal (δ, 3.3 ppm) from the mercaptothiazoline at the end of the PNIPAAm chain was visible in the ^1^H NMR spectrum. The molecular weight of the as-obtained PNIPAAm was determined by ^1^H NMR through comparing the integral of the proton of the CH signal at δ = 4.01 ppm in the repeat unit of NIPAAm (Mn 9600 g mol^−1^).

### 2.5. Coupling Au NCs with PNIPAAm

Mercaptopyridine-activated PNIPAAm was added to a stirred solution of luminescent Au NCs (10 mg in 10 mL of PBS buffer at pH 8.0). The mixed solution was stirred gently (300 rpm) for 24 h and then purified by a centrifugal filter (MWCO 10 kDa) to remove unreacted PNIPAAm and salts. After freeze-drying, the Au NCs-PNIPAAm conjugates were obtained.

## 3. Results and Discussion

### 3.1. Relay Luminescence Enhancement with Aggregation-Induced Emission (AIE)

In the present study, GSH-protected Au NCs were chosen as starting materials due to the following reasons: (i) GSH has various functional groups, such as thiol, amine, and carboxylic group. These functional groups can be used not only for the preparation of Au NCs, but also for the conjugation of other functional molecules; and (ii) GSH-protected Au NCs have high biocompatibility, which is a prerequisite for their applications in biomedicine [58,59,60]. Driven by these unique properties, GSH-protected luminescent Au NCs were synthesized according to the reported protocol [35]. To study the photothermal and thermosensitive properties of the as-synthesized luminescent Au NCs, it is important to functionalize the surface of the NCs with temperature-sensitive molecules. In this context, PNIPAAm is a good candidate because PNIPPAAm has a low critical solution temperature (LCST) of 32 °C. More interestingly, when the temperature is lower than LCST, it is hydrophilic (has good solubility in water), and when the temperature is higher than LCST, it becomes hydrophobic [61].

**Figure 1:**
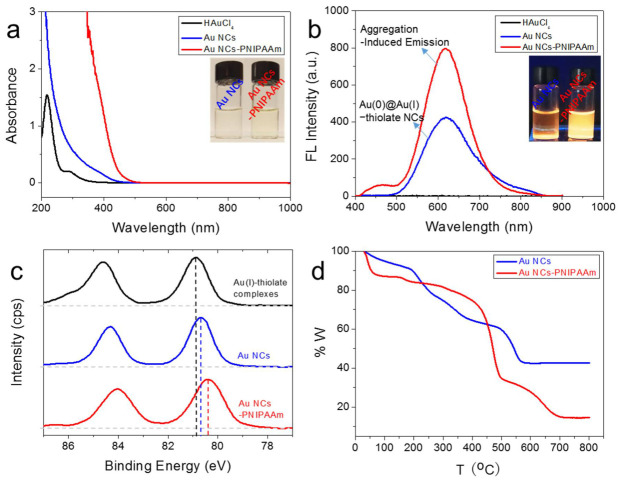
(**a**) UV-vis absorption and (**b**) photoemission spectra of HAuCl_4_, Au NCs, and Au NCs-PNIPAAm in aqueous solution (λ_ex_ = 365 nm, λ_em_ = 610 nm). (Insets) Digital photos of luminescent Au NCs and Au NCs-PNIPAAm under ambient light and UV light. (**c**) Au 4f XPS spectra of Au-thiolate complexes, luminescent Au NCs, and Au NCs-PNIPAAm. (**d**) TGA curve of Au NCs and Au NCs-PNIPAAm.

As a proof of concept, PNIPAAm was synthesized according to our reported protocol [62,63,64,65,66,67,68]. The as-generated mercaptothiazoline-activated PNIPAAm (Mn = 9600 g mol^−1^, PDI = 1.2, ca. monomer repeating units 85) was then conjugated to the primary amine on the surface of Au NCs (please refer to the experimental section for the detailed synthesis and characterizations, Figure S1). The resulting nanoconjugates (Au NCs-PNIPAAm) showed a significant change in hydrodynamic diameter from dynamic light scattering (DLS) analysis. Upon conjugation, the mean hydrodynamic diameter of the Au NCs with the polymer increased from 1.7 ± 0.2 nm to 4.8 ± 0.3 nm (Figure S2–S5). The corresponding zeta potentials of Au NCs and Au NCs-PNIPAAm are −7.6 and −17.3 mV, respectively (Figure S6). The increase in the negative charge of Au NCs-PNIPAAm is attributed to the decrease in the number of amine groups from the GSH ligands, which form amide bonds with PNIPAAm. In addition, unlike Au NCs, a broad peak at ~400 nm can be clearly seen from the UV-vis absorption spectrum of Au NCs-PNIPAAm (Figure 1a), which also supports the conjugation of PNIPAAm on the cluster surface. The luminescence spectrum of Au NCs has a peak at ~610 nm (Figure 1b, blue curve). The luminescence intensity is obviously enhanced (~2 times) upon the conjugation with PNIPAAm (Figure 1b, red curve). In fact, this increase in luminescence depends on the coupling between PNIPAAm and Au NCs. In particular, the amide bond between PNIPAAm and GSH on the NC surface restricts intramolecular rotation and vibration by rigidifying the ligand shell on the cluster surface. As a result, luminescence decay through the non-radiative channels is reduced, thereby enhancing the luminescence of Au NCs in the solution.

The as-synthesized Au NCs-PNIPAAm were further characterized by X-ray photoelectron spectroscopy (XPS) and thermogravimetric analysis (TGA) to study the chemical bond formation during the coupling process and the thermal stability of Au NCs-PNIPAAm, respectively. The XPS spectra in Figure 1c indicate that the oxidation state of Au in Au NCs-PNIPAAm (red line) is lower than that of Au-GSH complexes (black line) and luminescent Au NCs (blue line). The Au 4f spectrum of Au NCs-PNIPAAm has lower binding energies (84.1 and 80.4 eV) than luminescent Au NCs (84.3 and 80.7 eV) and Au-GSH complexes (84.6 and 80.9 eV). This is because the trithiocarbonate group of PNIPAAm has a strong combining capacity [11,69], which is capable of recombining Au(I) to form a higher conjugated structure. The thermal stability of Au NCs-PNIPAAm was assessed by TGA (Figure 1d). The degradation of PNIPAAm from the cluster surface was evident from the obvious weight loss after 400 ^o^C. This result indicates that Au NCs-PNIPAAm was structurally robust even at elevated temperatures.

**Figure 2:**
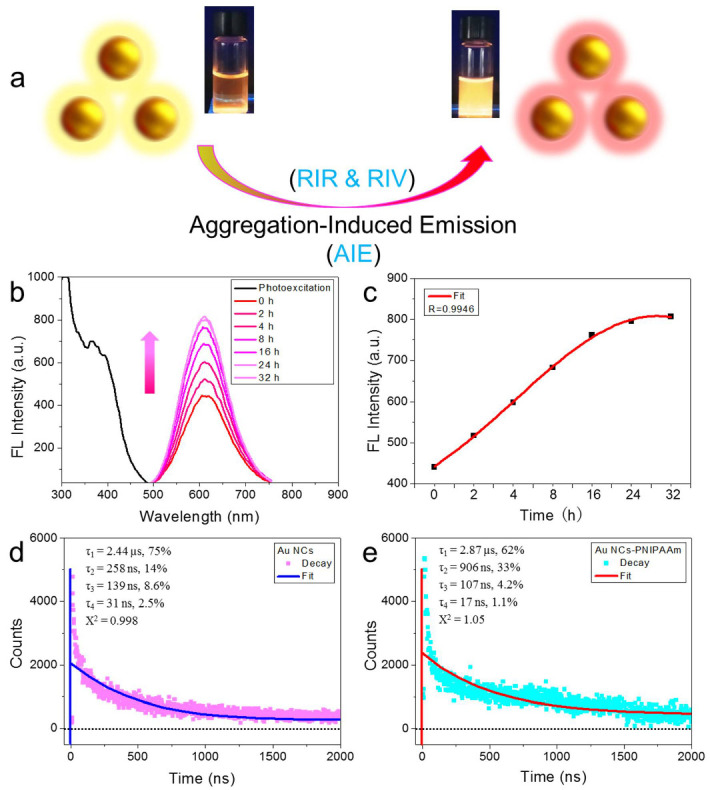
(**a**) Schematic illustration of the proposed mechanism for aggregation-induced emission (AIE), restriction of intramolecular rotation (RIR), and restriction of intramolecular vibration (RIV). (**b**) (Red lines) Photoemission spectra of the solution with different reaction times of the coupling process (the initiation of the reaction was defined as 0 h). (Black line) Photoexcitation spectra of Au NCs-PNIPAAm after 0 h of the coupling reaction. The excitation wavelength of the photoemission spectra is 400 nm and the emission wavelength of the photoexcitation spectrum is 620 nm. (**c**) The relationship between the luminescence intensity and the coupling time. (**d**,**e**) Photoluminescence decay profiles of the luminescent Au NCs and the as-synthesized Au NCs-PNIPAAm in water (0–2000 ns).

After successfully synthesizing Au NCs-PNIPAAm in aqueous solution, the effect of PNIPAAm ligand on the relay luminescence enhancement was studied (Figure 2a). In this case, we first monitored the synthesis process in a reaction time-dependent manner. As shown in Figure 2b,c, as time increased from 0 to 32 h, the luminescence peak intensity at ~610 nm (excitation wavelength at 365 nm) increased monotonically to 16 h and stabilized after 24 h, indicating that the combination process is step by step. The excited state lifetime decay profiles of the aqueous dispersion of Au NCs and Au NCs-PNIPAAm at λ = 610 nm (λ_ex_ = 344 nm) indicate the predominance of microsecond lifetime components in both the luminescent Au NCs (2.44 µs, 75%; 139 ns, 8.6%) and Au NCs-PNIPAAm (2.87 µs, 62%; 107 ns, 4.2%) (Figure 2d,e). The residuals of the two spectra are close to zero (Figure S7). The result corresponds to a relatively longer lifetime of Au NCs-PNIPAAm than Au NCs and explains the effect of relay luminescence enhancement. In particular, the PNIPAAm ligands were covalently attached to the surface of Au NCs and further induced the aggregation of Au NCs, which led to enhanced luminescence through RIR and RIV mechanisms. In addition, the trithiocarbonate group has a strong combining capacity and forms a larger conjugated structure through a recombining process with Au. Therefore, we attributed this interesting relay luminescence enhancement to the mechanism of RIR and RIV with AIE characteristics.

### 3.2. Relay Intensity Decrease with Temperature

**Figure 3:**
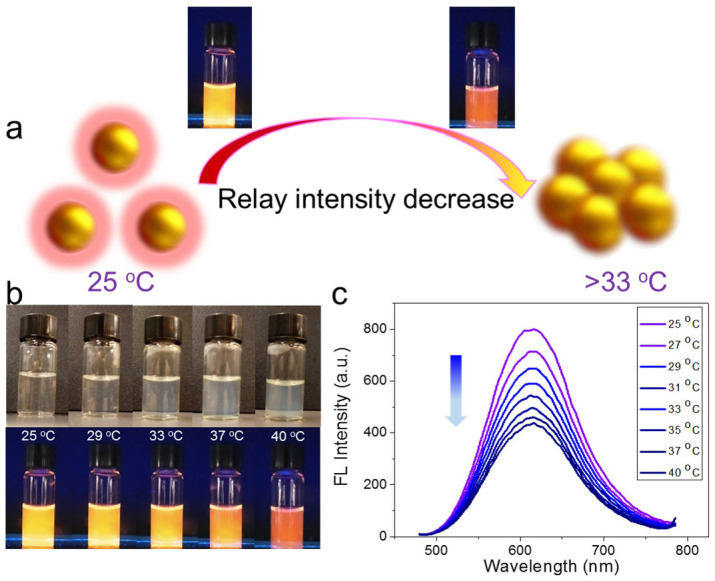
(**a**) Schematic illustration of the proposed mechanism for relay intensity decrease, where a higher temperature would induce the shrinkage of the thermosensitive polymer (PNIPAAm), leading to the weakened luminescence. (**b**) Digital photos of Au NCs-PNIPAAm (1 mg mL^−1^) in water with increasing temperature under visible (top row) and UV light (365 nm, bottom row). (**c**) The corresponding photoemission spectra of Au NCs-PNIPAAm at different temperatures (the excitation and emission wavelengths are 400 and 610 nm, respectively).

Considering the high luminescence of Au NCs, which is known to be sensitive to the environment, and the fact that the Au NCs were coupled with a thermoresponsive polymer, the thermoresponsive properties of Au NCs-PNIPAAm were studied. As shown in the digital photos of Figure 3b, the solutions of Au NCs-PNIPAAm gradually turned turbid with the increase of temperature at a constant conjugate concentration of 1 mg mL^−1^. In contrast, the corresponding luminescence was weakened, especially at temperatures above 32 °C (bottom row, Figure 3b). The opaque and weak luminescence of the solution strongly indicates the change of Au NCs-PNIPAAm with increasing temperature. As a proof of concept, when the temperature decreases, the luminescence of the corresponding Au NCs-PNIPAAm solution returns to the original intensity (Figure S8). However, it is undeniable that the luminescence intensity is not as high as before, which also indirectly proves that the luminescence of Au NCs-PNIPAAm solution has good cycling characteristics. Furthermore, different luminescence intensities can be cycled multiple times with temperature changes (Figure S9). It is worth mentioning that the thermoresponsive nature of luminescent Au NCs-PNIPAAm upon aggregation was different from the previously described relay luminescence enhancement.

Although in both cases aggregation plays an important role in the luminescence properties, the mechanism of temperature-induced luminescence changes seems to be consistent with the common intensity decrease with temperature. However, the temperature here causes changes in the optical properties of Au NCs, which is different from the conventional ACQ effect. To verify this, DLS measurements were performed, showing that the mean hydrodynamic diameter of Au NCs-PNIPAAm changed from 4.8 ± 0.3 nm at room temperature to 68.7 ± 1.0 nm at 37 °C (Figure S10). In particular, the increase in the hydrophobicity of PNIPAAm will cause the aggregation of Au NCs-PNIPAAm, which may be the main reason for the decrease of luminescence. The corresponding luminescence spectra clearly showed that when the solution temperature increased from 25 to 40 °C, the luminescence intensity of Au NCs-PNIPAAm gradually decreased (Figure 3c). The above results indicate that the change in temperature is crucial to the optical properties of Au NCs-PNIPAAm, that is, increasing temperature reduces the luminescence intensity of the composites. Therefore, this relay intensity decrease with temperature can provide a good platform to study the relay decay after the relay luminescence enhancement (Figure 3a).

### 3.3. In-Situ Observations of Relay Intensity Decrease

**Figure 4:**
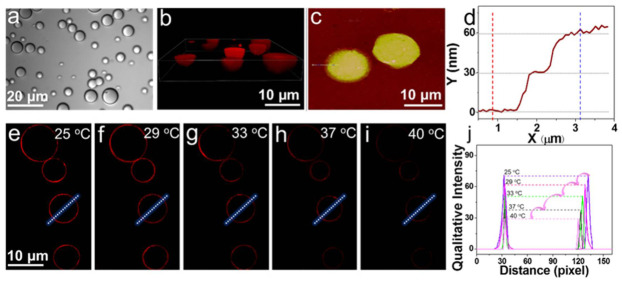
(**a**) Optical microscope image and (**b**) 3D CLSM image of Au NCs-PNIPAAm vesicles. (**c**) AFM images of the dry vesicles and (**d**) the corresponding section scanning of the films (red/blue line, the average value). (**e**–**i**) In-situ CLSM images of Au NCs-PNIPAAm vesicles from 25 to 40 °C showing the temperature-induced relay decrease in luminescence intensity accompanied by the decrease in diameter. (**j**) Corresponding intensity profiles and diameter variation for single vesicle shown in (**e**–**i**).

The relay properties provide a unique perspective for the functionalized Au NCs-PNIPAAm to evaluate the optical properties of Au NC derivatives. To quantitatively evaluate the changes in the relay intensity of the constructed Au NCs-PNIPAAm, we studied the self-assembly of Au NCs-PNIPAAm conjugates in solution. These conjugates can be used as building blocks of vesicles through a typical Pickering emulsion procedure. 2-ethyl-1-hexanol was used as the organic phase at an aqueous/organic volume fraction of 0.06. In the presence of the cross-linking agent glutaraldehyde (GA), the resulting vesicles can be transferred well into the aqueous solution without loss of structural integrity, which can be verified by luminescence microscope, confocal laser scanning microscope, transmission electron microscope (TEM), and atomic force microscope (AFM). The diameters of the constructed vesicles are in the range of 5–20 μm and their structures were found to be robust with non-aggregated status in aqueous solution evidenced by optical microscope (Figure 4a). Luminescence microscope images showed strong emission at the red channel, indicating that the as-prepared Au NCs-PNIPAAm has strong luminescence and high stability in aqueous solution (Figure S11). The optical and luminescence microscope images of the partially dried structures are composed of flexible ultrathin films that are visible to the naked eyes (Figure S12). TEM images of the vesicles suggest that the ultrathin films were at a different level, and the enlarged edges of the vesicles were continuous and integrated even under vacuum (Figure S13). In addition, through the section scanning model (Figure 4c,d), the AFM images of the vesicles showed different thicknesses, about 13 nm in different regions of the films, which is associated with the different level of the films.

To further reveal the structure of self-assembled vesicles, we studied the 3D structure in different ways. The 3D confocal laser scanning microscopy (CLSM) images (Figure 4b and Figure S14) showed the hollow structures that are robust without loss of structural integrity. The 3D AFM images (Figure S15) also suggest that the height of the vesicles is about 90 nm. By scanning the XY and YZ axes, the structure of the films can be clearly seen from the bottom and right images (Figure S16).

According to the difference in surface roughness, the films are structurally robust. More importantly, certain areas of the folded films are robust even when they are distorted to a great extent. Considering the unique thermosensitive characteristics of Au NCs-PNIPAAm, the above results suggest that the relay intensity is reduced due to temperature modulation. We also studied whether increasing the temperature will affect the surface roughness and the intact structure of the films. To confirm this, the corresponding roughness (Ra) decreased from 2.00 nm (25 °C) to 1.48 nm (37 °C) without/with increasing temperature, which is associated with a temperature higher than LCST (32 °C), indicating that the thermosensitive PNIPAAm remained attached to the films (Figure S17). Inspired by the above results, to further understand whether the temperature-induced optical property transition will affect the vesicles in the aqueous solution, we attempted to verify the continuous heating effects. Indeed, we found that the increase in temperature was also responsible for the reduction in size. As shown in Figure 4e–i, the temperature stimuli behavior was monitored using CLSM by measuring individual water-dispersed vesicles thermally cycling from 25 to 33 °C. As the temperature increased, the phase change of PNIPAAm resulted in about 58.6% of luminescence intensity loss and 9% of decrease in the vesicle diameter (Figure 4j). The generated Au NCs-PNIPAAm vesicles showed obvious changes in optical properties against temperature-induced relay intensity decrease.

## 4. Conclusions

Luminescent Au NCs were functionalized with thermosensitive PNIPAAm through covalent bonds, showing two interesting phenomena, namely, relay luminescence enhancement by AIE and temperature-induced relay intensity decrease. The restriction of intramolecular rotation and vibration and the formation of higher conjugated structures, both induced by PNIPAAm, resulted in relay luminescence enhancement. On the other hand, PNIPAAm on the cluster surface became denser at a temperature higher than LCST, resulting in relay intensity decrease. This relay intensity decrease can also be achieved by assembling Au NCs-PNIPAAm into vesicles. The findings revealed in this study may help to explore the optical properties of metal NCs in various applications.

## Data Availability

Data is contained within the article or in Electronic Supporting Information (ESI).

## Supplementary Materials

The following are available online at https://www.mdpi.com/article/10.3390/nano12050777/s1, Figure S1. ^1^H NMR spectrum of the end-capped mercaptothiazoline-activated PNIPAAm (Mn 9600 g mol^−1^) in CDCl_3_, 400 MHz; Figure S2: DLS profiles of the aqueous Au NCs (black, 1.7 ± 0.2 nm) and Au NCs-PNIPAAm (red, 4.8 ± 0.3 nm) in water at room temperature; Figure S3. A representative TEM image of the as-synthesized luminescent Au NCs; Figure S4. Particle size distribution of the aqueous Au NCs (1.7 ± 0.2 nm) in water at room temperature; Figure S5. Particle size distribution of the aqueous Au NCs-PNIPAAm (4.8 ± 0.3 nm) in water at room temperature; Figure S6. Zeta potential measurement for Au NCs (purple, −7.6 mV) and Au NCs-PNIPAAm (red, −17.3 mV) in water at room temperature; Figure S7. (a, b) Photoluminescence decay profiles of Au NCs and Au NCs-PNIPAAm (0–8000 ns) in water. (c, d) Residuals of the corresponding lifetime decay profiles of Au NCs and Au NCs-PNIPAAm; Figure S8. Digital photos of Au NCs-PNIPAAm (1 mg mL^−1^) in water with decreasing temperature under UV light (365 nm); Figure S9. Luminescence intensity of five complete reversible cycles as followed by alternate low temperature (25 °C) and high temperature (37 °C); Figure S10. Size distribution of Au NCs-PNIPAAm below (red, 4.8 ± 0.3 nm, 25 °C) and above (blue, 68.7 ± 1 nm, 37 °C) the LCST of PNIPAAm; Figure S11. Representative fluorescence microscope image of the Au NCs-PNIPAAm vesicles; Figure S12. (a) Optical microscope and (b) fluorescence microscope image of dry Au NCs-PNIPAAm vesicles; Figure S13. Transmission electron microscope (TEM) images of Au NCs-PNIPAAm vesicles (left), and the enlarged region (right), showing the structure of the films at different levels; Figure S14. CLSM images of the 3D structure of the Au NCs-PNIPAAm vesicles; Figure S15. Atomic force microscope (AFM) images of Au NCs-PNIPAAm vesicles, and the clear stereoscopic structure showing the distinguishing height from the edge to center; Figure S16. 3D scanning model of the XY and YZ axes, showing the continuous and robust films of the constructed Au NCs-PNIPAAm vesicles; Figure S17. Atomic force microscope (AFM) images of Au NCs-PNIPAAm vesicles: (a) below and (b) above the LCST, showing the different roughnesses of the selected regions a (Rq = 2.54 nm, Ra = 2 nm) and b (Rq = 1.87 nm, Ra = 1.48 nm). The following data corresponds to the roughness parameter of the film.
