# Supplementary material for: Engineering Au Nanoclusters for Relay Luminescence Enhancement with Aggregation-Induced Emission
Source: Nanomaterials (Basel). 2022 Feb 25;12(5):777. doi: 10.3390/nano12050777 (PMC8912310; doi:10.3390/nano12050777)
Supplement: Supplementary file 1 [file nanomaterials-12-00777-s001.zip › nanomaterials-1534694-supplementary.pdf]

nanomaterials

Supplementary Materials

Engineering Au Nanoclusters for Relay Luminescence Enhancement with Aggregation-Induced Emission

Pei Zhou [1,2,*], Nirmal Goswami [3], Tiankai Chen [2], Xiaoman Liu [4] and Xin Huang [4,*]

[1] School of Environmental and Municipal Engineering, North China University of Water Resources and Electric Power, Collaborative Innovation Center for Efficient Utilization of Water Resources, Henan Key Laboratory of Water Environment Simulation and Treatment, Zhengzhou 450046, China
[2] Department of Chemical and Biomolecular Engineering, National University of Singapore, 10 Kent Ridge Crescent, 119260, Singapore; chen.tiankai@u.nus.edu
[3] Materials Chemistry Department, CSIR-Institute of Minerals and Materials Technology, Acharya Vihar, Bhubaneswar, Odisha 751013, India; ngoswami@immt.res.in
[4] MIIT Key Laboratory of Critical Materials Technology for New Energy Conversion and Storage, School of Chemistry and Chemical Engineering, Harbin Institute of Technology, Harbin 150001, China; liuxiaoman@hit.edu.cn
* Correspondence: peizhou@ncwu.edu.cn (P.Z.); xinhuang@hit.edu.cn (X.H.)

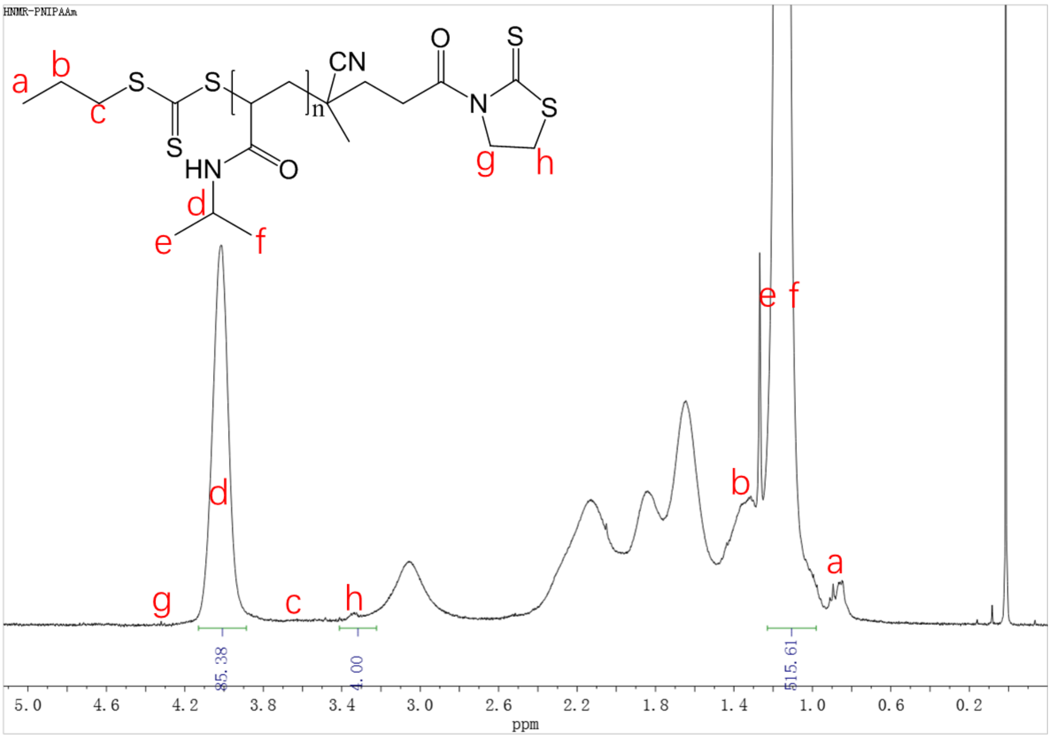

Figure S1. ^{1}H NMR spectrum of the end-capped mercaptothiazoline-activated PNIPAAm (Mn 9600 g mol^{-1}) in $CDCl_3$, 400 MHz.

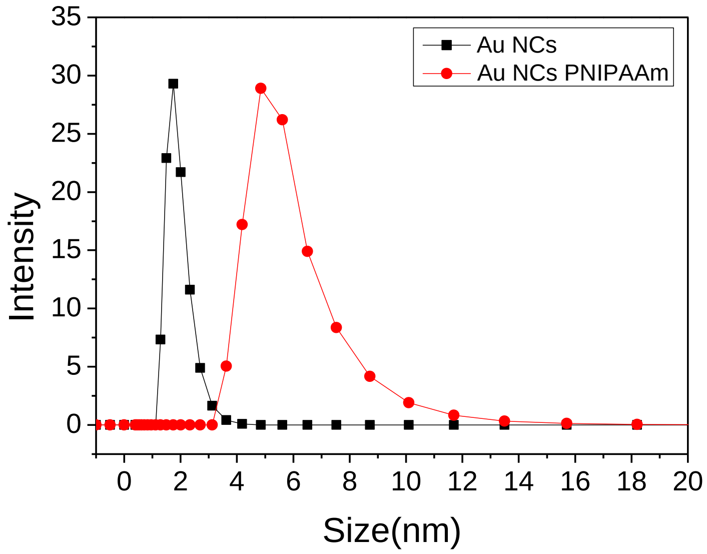

Figure S2. DLS profiles of the aqueous Au NCs (black, 1.7 ± 0.2 nm) and Au NCs-PNIPAAm (red, 4.8 ± 0.3 nm) in water at room temperature.

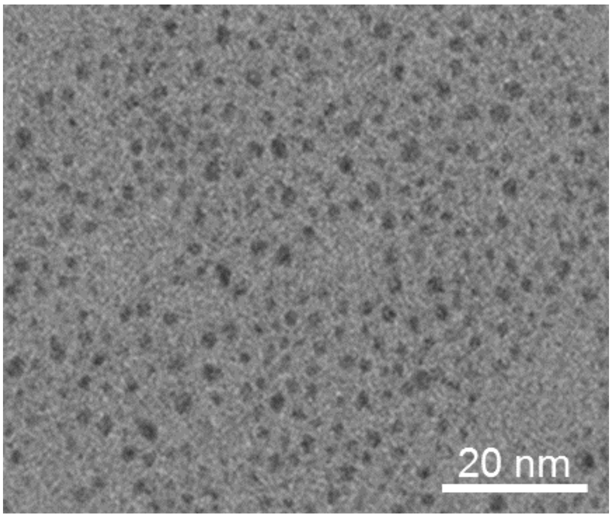

Figure S3. A representative TEM image of the as-synthesized luminescent Au NCs.

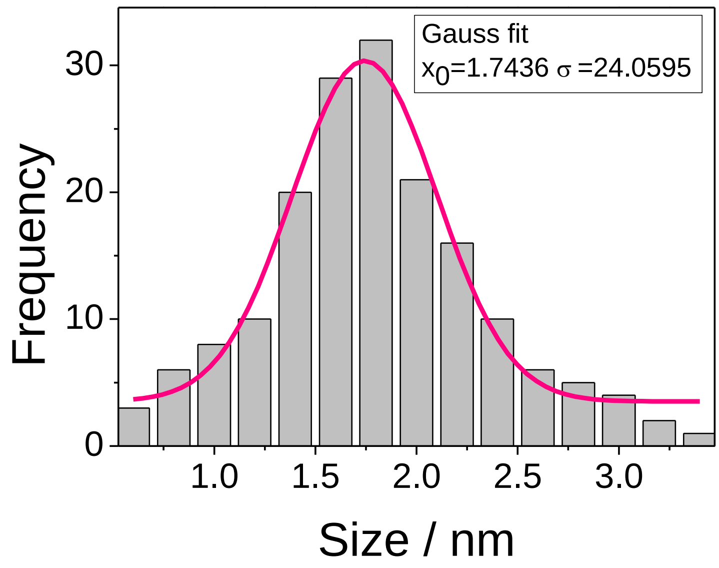

Figure S4. Particle size distribution of the aqueous Au NCs (1.7 ± 0.2 nm) in water at room temperature.

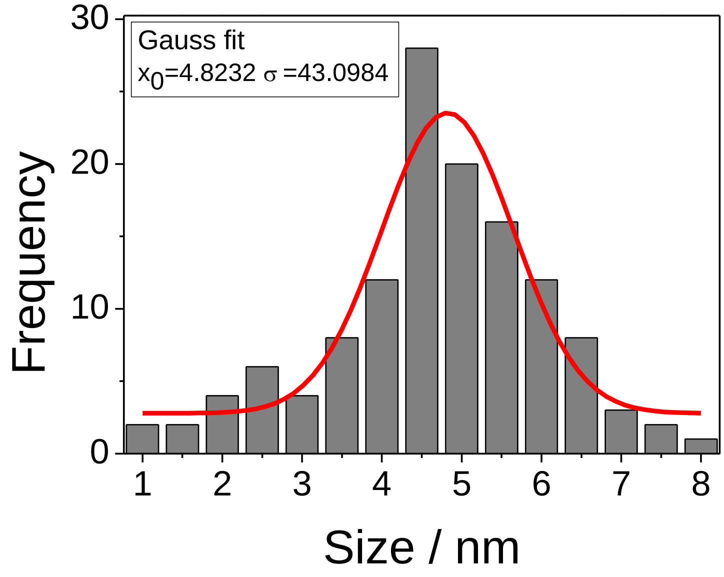

Figure S5. Particle size distribution of the aqueous Au NCs-PNIPAAm (4.8 ± 0.3 nm) in water at room temperature.

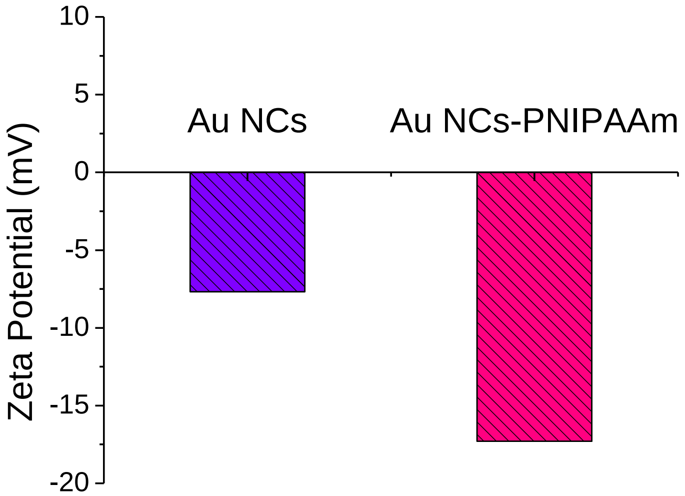

Figure S6. Zeta potential measurement for Au NCs (purple, −7.6 mV) and Au NCs-PNIPAAm (red, −17.3 mV) in water at room temperature.

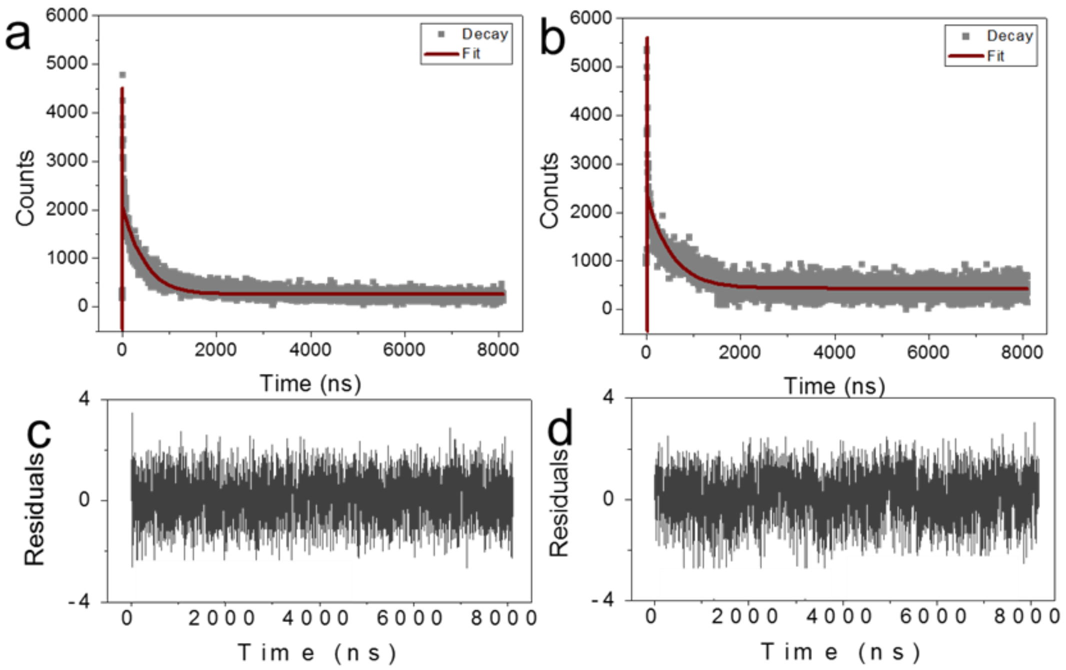

Figure S7. (**a**,**b**) Photoluminescence decay profiles of Au NCs and Au NCs-PNIPAAm (0–8000 ns) in water. (**c**,**d**) Residuals of the corresponding lifetime decay profiles of Au NCs and Au NCs-PNIPAAm.

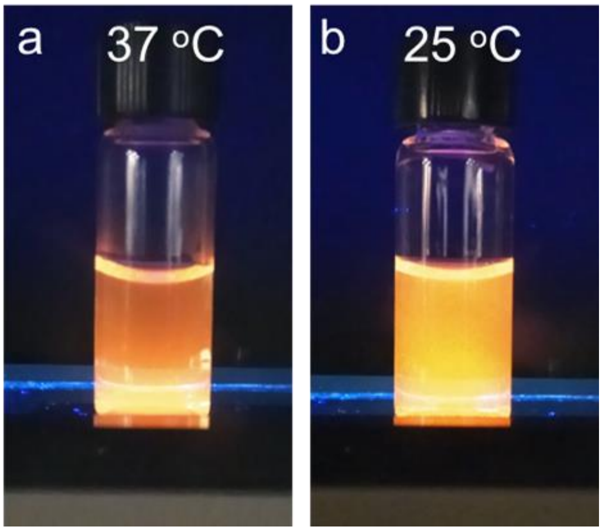

Figure S8. Digital photos of Au NCs-PNIPAAm (1 mg mL-1) in water with decreasing temperature under UV light (365 nm) (a, above LCST, b, below LCST).

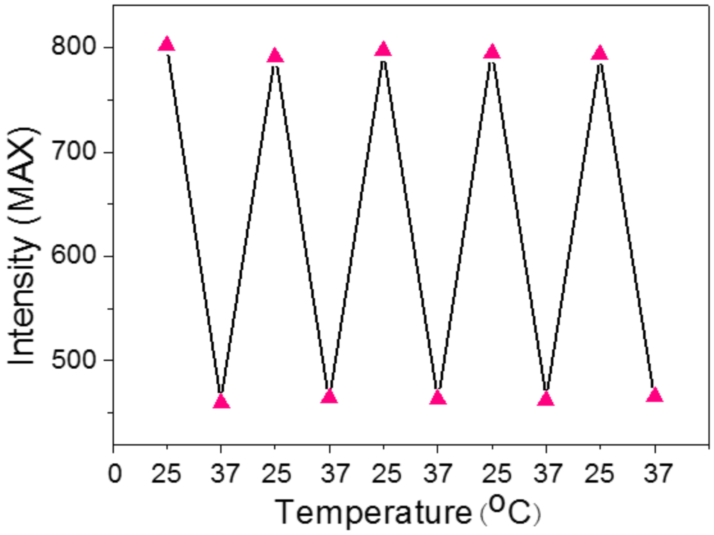

Figure S9. Luminescence intensity of five complete reversible cycles as followed by alternate low temperature (25 °C) and high temperature (37 °C).

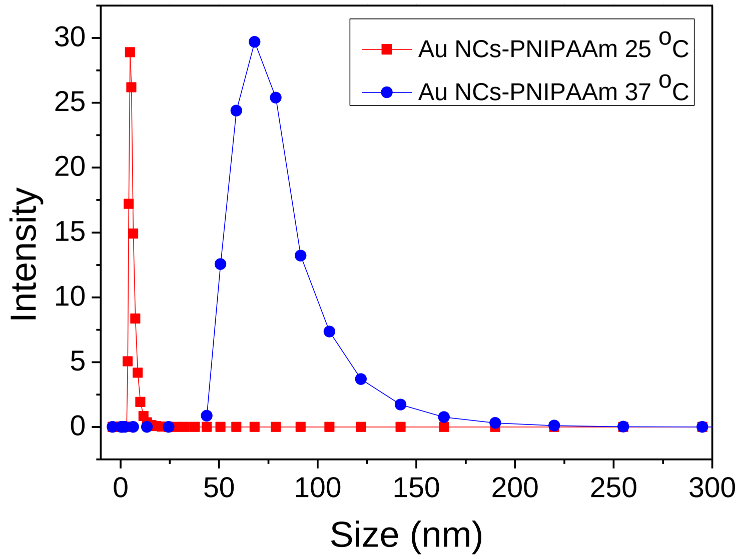

Figure S10. Size distribution of Au NCs-PNIPAAm below (red, 4.8 ± 0.3 nm, 25 °C) and above (blue, 68.7 ± 1 nm, 37 °C) the LCST of PNIPAAm.

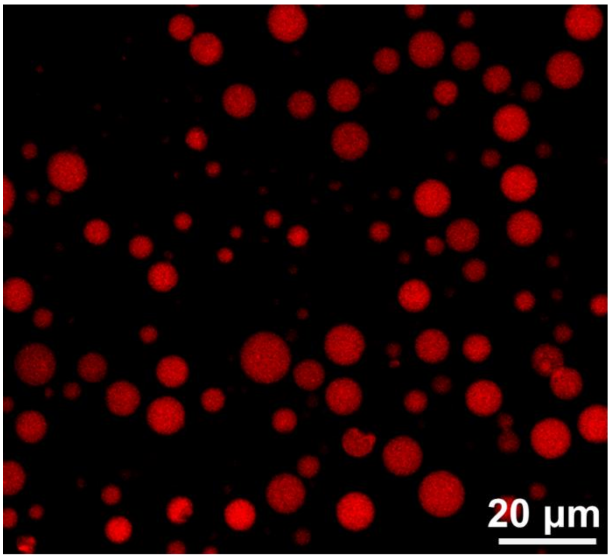

Figure S11. Representative fluorescence microscope image of the Au NCs-PNIPAAm vesicles.

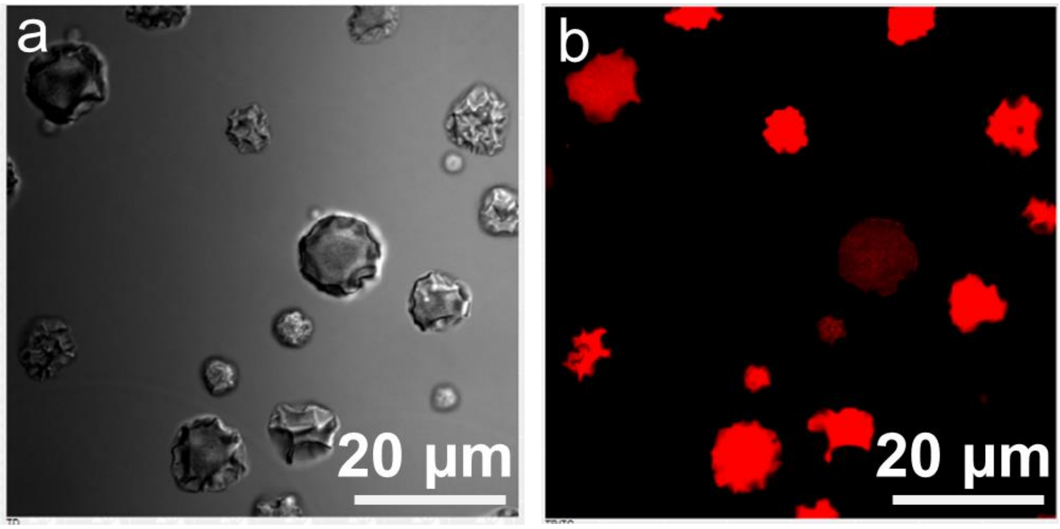

Figure S12. (**a**) Optical microscope and (**b**) fluorescence microscope image of dry Au NCs-PNIPAAm vesicles.

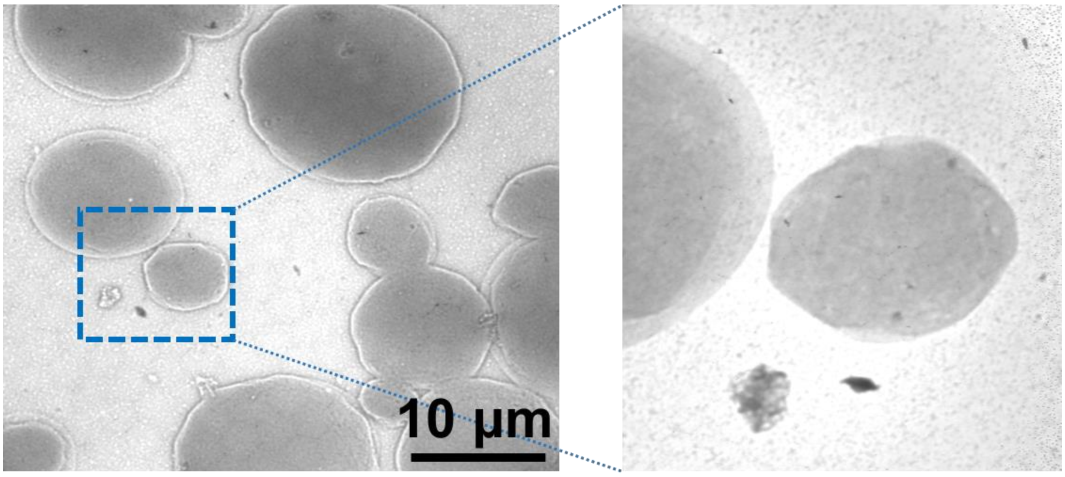

Figure S13. Transmission electron microscope (TEM) images of Au NCs-PNIPAAm vesicles (left), and the enlarged region (right), showing the structure of the films at different levels.

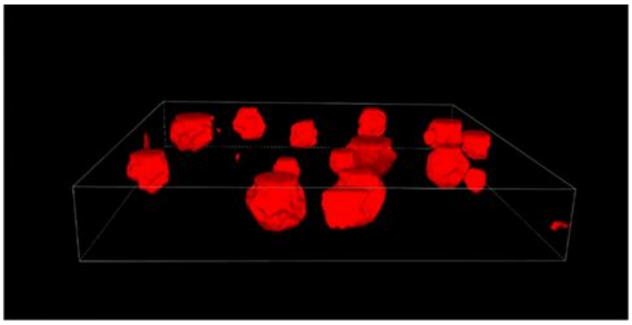

Figure S14. CLSM images of the 3D structure of the Au NCs-PNIPAAm vesicles,.

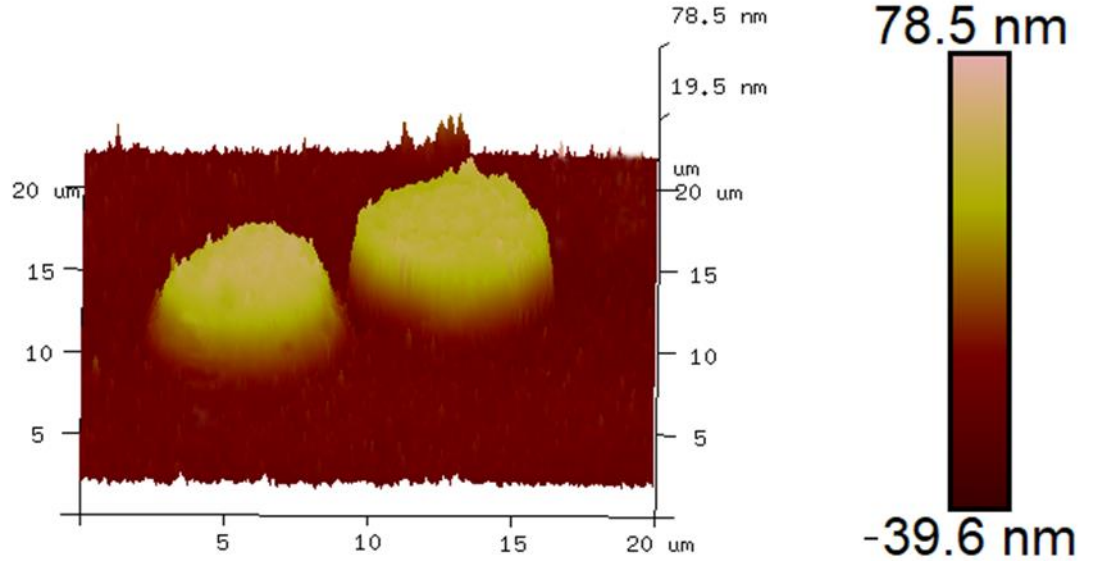

Figure S15. Atomic force microscope (AFM) images of Au NCs-PNIPAAm vesicles, and the clear stereoscopic structure showing the distinguishing height from the edge to center.

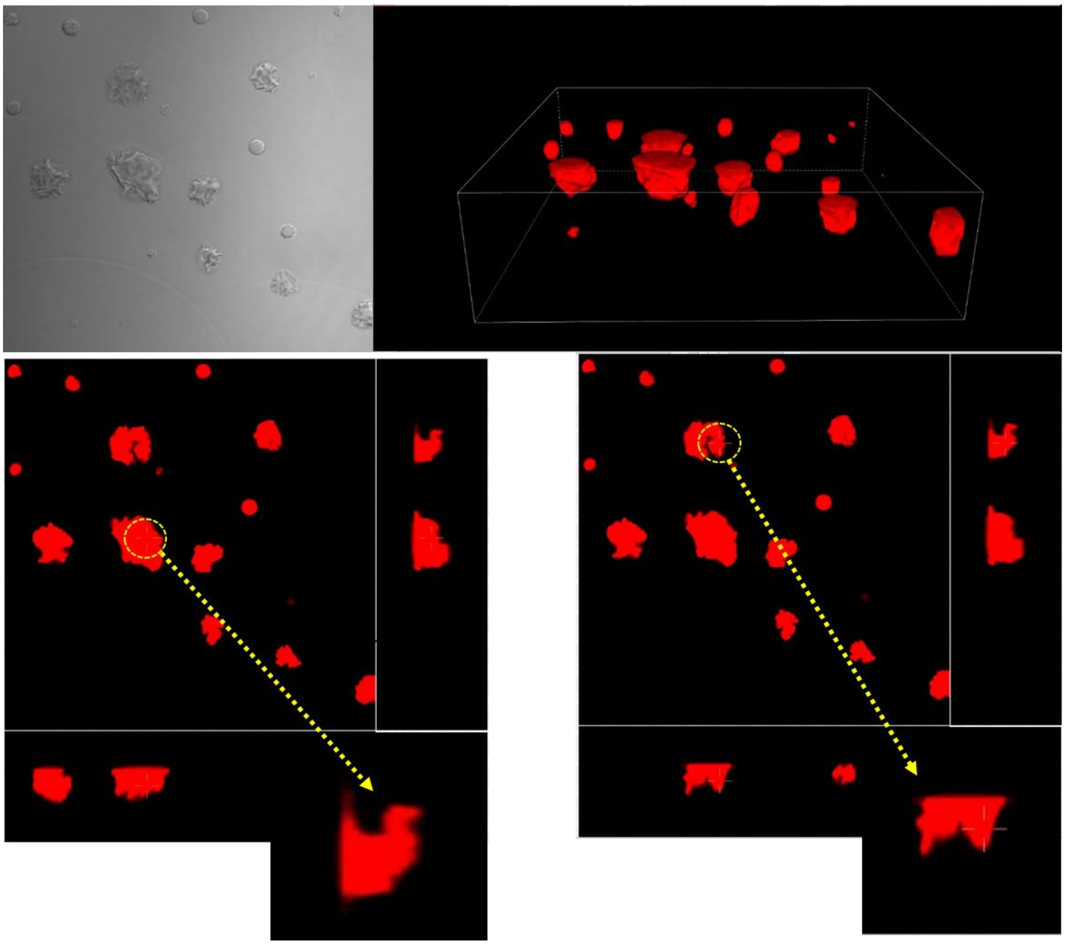

Figure S16. 3D scanning model of the XY and YZ axes, showing the continuous and robust films of the constructed Au NCs-PNIPAAm vesicles (left/right, approach the films from different angles).

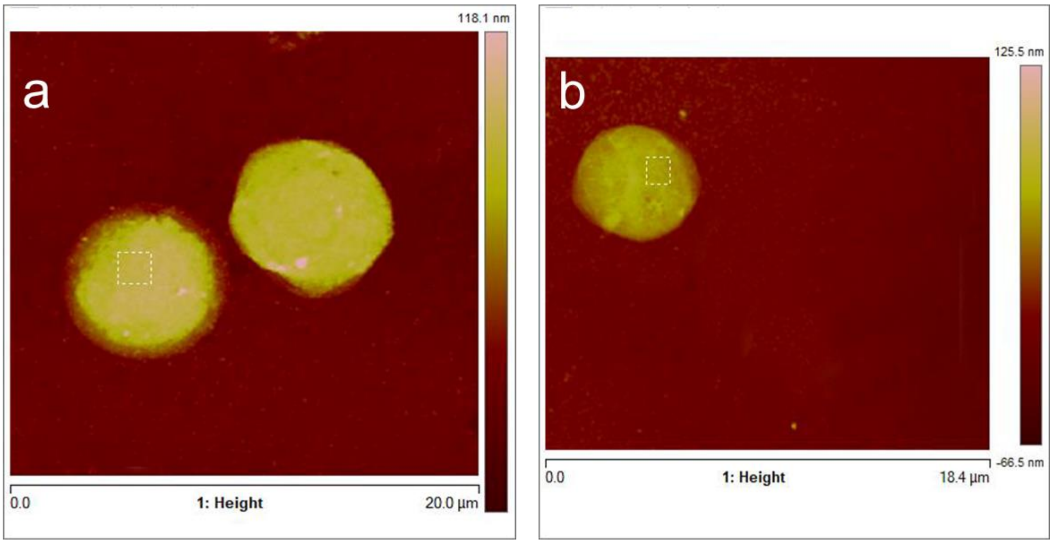

Rq	2.54 nm	Rq	1.87 nm
Ra	2.00 nm	Ra	1.48 nm

Figure S17. Atomic force microscope (AFM) images of Au NCs-PNIPAAm vesicles: (**a**) below and (**b**) above the LCST, showing the different roughness of the selected regions a (Rq = 2.54 nm, Ra = 2 nm) and b (Rq = 1.87 nm, Ra = 1.48 nm). The lower portion of the corresponding roughness parameter of the film.
